# Effects of squatting with elastic bands or conventional resistance-training equipment at different effort levels in post-exercise intraocular pressure of healthy men

**DOI:** 10.5114/biolsport.2022.109955

**Published:** 2021-11-10

**Authors:** Javier Gene-Morales, Andrés Gené-Sampedro, Rosario Salvador-Palmer, Juan C. Colado

**Affiliations:** 1 Research Unit in Sport and Health, University of Valencia, Valencia, Spain; 2 Research Institute on Traffic and Road Safety (INTRAS), University of Valencia, Valencia, Spain; 3 Research Group in Prevention and Health in Exercise and Sport, University of Valencia, Valencia, Spain; 4 Department of Optics and Optometry and Vision Sciences of the University of Valencia, Valencia, Spain; 5 Department of Physiology of the University of Valencia, Valencia, Spain

**Keywords:** Variable resistance, Strength exercise, Ocular perfusion pressure, Central corneal thickness, Cardiovascular, Blood pressure

## Abstract

This study aimed to compare intraocular pressure (IOP), mean ocular perfusion pressure (MOPP) and central corneal thickness (CCT) acute adaptations to squat exercise using elastic bands (EB) or weight plates (WP) together with the weight of the bar and applying maximal or submaximal efforts. Cardiovascular parameters (pulse pressure, mean blood pressure, heart rate), rate of perceived exertion, kilograms, and number of repetitions served to monitor psychophysiological acute variations. Twenty physically active males (25.55 ± 4.75 y.o.) underwent two sessions (one for familiarization and one for the experimental trial). In the experimental session, ocular and cardiovascular pre-exercise measurements were taken. Then, two sets using WP and two using EB attached to the bar with the same load were performed by each subject in random order. Immediately after finishing each set, the subjects rated perceived exertion, and cardiovascular and ocular measurements were taken, in this order. An ANOVA with post-hoc LSD evaluated differences between sets. IOP significantly decreased (*p* < 0.001, ƞp^2^ = 0.513), and MOPP (*p* < 0.001, ƞp^2^ = 0.413) and cardiovascular variables significantly increased due to the exercise effect; CCT changes were non-significant. No significant effect of the material, level of effort, or their interaction was observed in the IOP and MOPP (*p* > 0.05). EB permitted more repetitions to be performed and led to non-significantly lower post-exercise IOP values (effect size [*d*] compared to resting 0.79 and 1.00) in comparison to WP (*d* = 0.73–0.74). IOP and ocular and systemic hemodynamic responses are similar when using EB instead of WP to load the bar, with EB allowing a larger number of repetitions. Data presented in this paper may help with the prescription of resistance training for people with glaucoma risk factors.

## INTRODUCTION

Resistance exercise has been shown to acutely influence parameters of ocular physiology which are strongly related to ocular health, specifically ocular perfusion pressure (OPP) and intraocular pressure (IOP) [1–4]. OPP represents the blood flow entering the eye, which is, in turn, conditioned by the IOP [5]. There is still no consensus on whether resistance exercise acutely increases [6–10] or decreases IOP [11–17]. To the best of our knowledge, only one study has analysed IOP adaptations after four weeks of an upper-body resistance-exercise programme using loads between 40 and 60% of one-repetition maximum (1RM) and found significant decreases in basal values [18].

Numerous devices can be used to enhance the positive effects of resistance exercise [19]. Specifically, elastic bands (EB) are increasing in popularity for both health and physical performance [20–22] due to inducing similar adaptations to traditional resistances [23, 24] and providing optimal resistance across the entire range of motion when EB are attached to the bar for squat exercises (reduced weight during the lowest phases of the range of motion and increased at the upper phases) [25–27]. As far as we know, no previous research has investigated the different acute adaptations that may occur in ocular parameters when using EB instead of weight plates (WP) to load the bar in squats.

It is important to understand that in the deepest phases of the squat a large increase in difficulty occurs, which increases the effort of the core and respiratory muscles [28]. This is related to augmented intrabdominal and intrathoracic pressure [28] and may decrease the venous return through the vena cava. This increases the choroidal volume and episcleral venous pressure and thereby ensures an elevation in IOP [1, 2]. Given that the EB reduce the load throughout this biomechanically disadvantageous region of the movement [26] due to their elongation coefficient [25], the question arises whether the use of EB in resistance exercises could generate different responses in ocular parameters compared to conventional resistance (e.g., weight plates).

The purpose of this study was to analyse IOP, mean ocular perfusion pressure (MOPP), and other ocular health-related parameters after a squat exercise protocol using elastic bands or weight plates to load the bar and performing maximal or submaximal efforts. Bearing in mind the weight reduction that happens with elastic bands in the lower phases of the squat movement, we hypothesize that this material will allow for lower IOP values and higher MOPP than weight plates.

## MATERIALS AND METHODS

This quasi-experimental study assessed the changes in ocular health-related parameters after performing squats on a Smith machine considering the use of WP or EB to load the bar and maximal or sub-maximal efforts. Besides the main variables (IOP and MOPP), central corneal thickness (CCT) was measured to assess whether this parameter may condition IOP behaviour [3,29]. Rate of perceived exertion (RPE), heart rate (HR), mean blood pressure (MBP), and pulse pressure (PP) were used to characterize the cardiovascular adaptations to the exercise [19]. The four squat sets were as follows:

*Sets that used weight plates*: [1] maximum number of repetitions with 75% of one repetition maximum (1RM); [2] 9 repetitions (sub-maximal) with 75%1RM.

*Sets that used elastic bands*: [3] maximum number of repetitions with 75%1RM; [4] 10 repetitions (submaximal) with 75%1RM load.

A percentage of 1RM commonly employed in resistance training (75%1RM) was used [30, 31]. The pertinent kilograms were added to the bar with WP (28 mm cast iron plates from 0.50 to 20 kg; Domyos, France) or EB (looped CLX elastic bands; TheraBand, Akron, OH, USA) at the standing position of each subject. A researcher weighed the bar at this point using a 100 g precision scale (9179 SV3R; Salter, United Kingdom) to ensure the weight for each set with EB. Repetitions for the submaximal sets (Sets 2 and 4) were established at 9 with WP and 10 with EB due to observing that subjects could perform 10 or more repetitions with WP and many more than 10 repetitions with EB in the pilot studies.

### Subjects

A sample size of 19 participants was determined by a power analysis (G*Power 3.0; [32]) assuming an α of 0.05, a power level of 0.8, an effect size of f(V) = 0.87 as obtained in the pilot studies, and a non-sphericity correction of e = 1. Inclusion criteria were: 1) younger than 40 years, 2) experience with strength training for at least 6 months and performing at least 2 days per week of lower limb training including squats, 3) no musculoskeletal health issues, 4) normal visual health, and 5) no history of ocular disease or surgery. All participants had a baseline IOP ≤ 21 mmHg, systolic blood pressure (SBP) between 90 and 140 mmHg, diastolic blood pressure (DBP) between 60 and 90 mmHg, and PP ≤ 65 mmHg, excluding possible cardiovascular and ocular disorders [33, 34].

As a result, of the 25 recruited, only 20 physically active males met the criteria and voluntarily participated in this study (mean age: 25.55 ± 4.75 years, 95% confidence interval (CI) [23.84–28.00]; body mass: 75.67 ± 9.02 kg, 95% CI [71.45–79.89]; body mass index (BMI): 24.04 ± 2.11 kg/m^2^, 95% CI [23.20–25.08]; body fat: 10.19 ± 2.29%, 95% CI [9.32–11.38]; squat 1RM: 126.53 ± 24.62 kg, 95% CI [116.32–138.26]; ratio 1RM-body-weight (relative strength): 1.68 ± 0.35, 95% CI [1.53–1.84]). All participants were instructed to avoid alcohol consumption and vigorous exercise for 24 h before any of the sessions. They were asked to sleep for at least 8 h, not to consume stimulants or smoke, not to drink more than 1 L of liquids [2], and not to perform prolonged near-viewing activities in the 3 h before the trials [35].

### Procedures

All measurements were conducted in the same laboratory by the same researchers (one optometrist, and two sports scientists) at the Optometric Clinic “Fundació Lluís Alcanyís” from the University of Valencia (Spain). All data were collected in a thermoneutral environment (˜22ºC and ˜60% humidity), under the same lighting, and between 10:00 h and 13:00 h as the IOP is more stable within this time period [3]. One session for assessment and familiarization and one experimental trial to evaluate all dependent variables were carried out separated by 48 h. Both sessions lasted around 90 minutes.

In the familiarization session, the participants signed the consent form and filled in the demographic questionnaire and guarantee of data confidentiality. They also underwent a physical and visual examination to ensure they complied with the inclusion criteria and to assess their squat technique. Anthropometric measurements were obtained with a height rod (IP0955; Invicta Plastics Limited, Leicester, England) and a bioelectrical impedance scale (Body Composition Analyzer BF-350; Tanita Corporation, Tokyo, Japan). BMI was calculated as weight [kg]/(height [m])^2^. At this point, the standardized warm-up was started, including joint mobility, bodyweight exercises (including squats), jogging, and dynamic stretching. As a part of the specific warm-up, participants were instructed on how to perform the squats at the correct movement tempo (see “Squat exercise” section) and how to use the RPE scales [36, 37]. After the warm-up, participants performed the RM testing. Before the testing, three approximation sets were performed: first, one set of 20 repetitions without additional weight (the bar weighed 20 kg), and then two more sets of 15 and 12 submaximal repetitions, out of 20 and 15 RM, respectively. Loads for these submaximal sets were selected according to the participant’s self-perception and researcher experience. Finally, a fatigue test with submaximal loads using WP was carried out at the study-specific squat tempo. This procedure consists of performing between 8 and 12 maximal repetitions and introducing the repetitions performed and the kilograms used in a formula [38–41]. If a participant performed more than 12 or less than 8 repetitions, the load was modified, and another set was performed. O’Connor or Brzycki formulas were used to obtain the load for 1RM. After appropriate rest, the RM obtained in the formula was tested and adjusted if necessary. Percentages were calculated for 75%1RM. At least a five-minute rest was allowed between sets; more time was permitted depending on the perception of the subjects [42].

At the beginning of the second session, baseline measurements of each variable were taken. After the warm-up, each set was performed in a random order (https://www.random.org/lists/). Immediately after the exercise, the researcher in charge recorded the number of repetitions and subjects reported RPE (less than 5 seconds) while sitting to undergo cardiovascular direct measurements (SBP, DBP, and HR; 30–40 seconds). Ocular measurements (IOP and CCT) were uniformly started 60 seconds after finishing each set. The physiological measurements were carried out in an adjacent space separated from the Smith machine by a partition screen to blind the researcher in charge. At least a five-minute rest was given between sets and water intake was avoided to prevent IOP changes due to hydration [1]. Constant feedback was used in both sessions to ensure proper execution and to encourage maximum effort [43].

### Squat exercise

A high-bar back squat to a parallel depth [22,44] was performed in a Smith machine (Multipower Smith Machine Powerline PSM144X; Body-Solid, USA). The stance width was established between the hips and shoulders. Shoes were used but no weightlifting belts or knee wraps were permitted. The tempo consisted of an inhalation-coordinated eccentric phase lasting two seconds [45] with a pause of one second at the deepest point and an exhalation-coordinated maximum speed concentric phase (4 seconds for a complete squat). The Valsalva manoeuvre was avoided due to its influence in IOP [1–4] by asking participants to perform audible breathing. A metronome (Metronome Beats v.4.4.0, Stonekick, London, England) was used at 60 bpm to standardize the movement tempo. The depth was adjusted with a horizontal elastic band. The participant had to touch the band (midthigh) in every repetition.

### Ocular measurements

IOP and CCT were measured in mmHg and microns, respectively, with a validated non-contact tonometer [46,47]. The Auto Kerato-Refracto-Tonometer TRK-1P (Topcon, Japan) automatically compensates for the corneal thickness. The intraclass correlation coefficient (ICC) of the measurements was 0.91 (coefficient of variation (CV)=6.24%) for the IOP and 0.93 (CV=0.95%) for the CCT. Measurements were taken in both eyes in the pilot study. Right eye measurements were used since no significant difference was observed between the eyes.

MOPP was calculated in mmHg using the formula MOPP=2/3(MBP)– IOP [4,34]. The results showed an ICC of 0.89 and a CV of 6.68%.

### Cardiovascular measurements

Cardiac measurements (SBP, DBP, and HR) were performed using a digital automatic blood pressure monitor (M6W HEM-7213-E (V), Omron, Japan) with an ICC of 0.90 and CV of 5.49% for the SBP, 0.86 and 3.77% for the DBP, and 0.91 and 6.05% for the HR. PP (mmHg) was calculated as SBP–DBP, and MBP (mmHg) as DBP+1/3(PP) [34].

### Perceived effort measurements

RPE was measured with the OMNI-RES [35] and OMNI-RES for EB [36]. Previous studies can be consulted for further details on how to apply these scales [47–49].

RPE was measured with the OMNI-RES [36] and OMNI-RES for EB [37]. Previous studies can be consulted for further details on how to apply these scales [48–50].

### Ethics

The study was conducted in conformity with the Code of Ethics of the World Medical Association (Declaration of Helsinki). Permission was provided by the Ethics Committee on Human Research of the University of Valencia (H1499867368458). Data reported in the present study form part of a research project investigating different ways of applying elastic resistance in squat performance. Previous data from this project have already been published [22, 43]. All participants voluntarily agreed to participate and were free to withdraw from the study at any time. They signed an informed consent form including a guarantee of data confidentiality.

### Statistical analyses

Statistical analyses were performed using commercial software IBM SPSS Statistics for Macintosh (Version 26.0; IBM Corp., Armonk, NY). The normality of data distribution was assessed using the Shapiro-Wilk test. All physiological variables showed a normal Gaussian distribution (*p* > 0.05). RPE followed a non-normal distribution (*p* ≤ 0.05).

To assess differences and evaluate the influence of the material and the type of effort on the dependent variables, two approaches were employed: 1) a two-way ANOVA with the material (EB and WP) and the level of effort (maximal and submaximal) as the within-subject factors and, 2) an analysis of variance (ANOVA) of repeated one-way measurements to evaluate differences between the resting values and the exercise sets. All the cases complied with Mauchly’s sphericity assumption. The effect size was evaluated with eta partial squared (ƞp^2^), where 0.01 < ƞp^2^ < 0.06 constitutes a small effect, 0.06 ≤ ƞp^2^ ≤ 0.14 medium, and ƞp^2^ > 0.14 a large effect. Pairwise post-hoc comparisons were carried out using the least significant difference correction (LSD). The effect size was calculated as Cohen’s *d* with Hedges corrections [51]. This value is reported as unbiased Cohen’s *d* (*d*_unb_) [52], with *d*_unb_ < 0.50 constituting a small effect, 0.50 ≤ *d*_unb_ ≤ 0.79 moderate, and *d*_unb_ ≥ 0.80 a large effect [53]. The test-retest relative reliability of the instruments was assessed in a subsample of 10 subjects (2 measurements per subject) calculating the ICC [54]. ICC was interpreted as poor (< 0.40), moderate (0.40–0.59), good (0.60–0.79) or excellent (≥ 0.80) [55]. The absolute reliability was verified with the coefficient of variation (CV) using the formula: (standard error of measurement (SEM)/mean of both measurements)x100; SEM is the standard deviation of the difference between the two measurements divided by the square root of the number of measurements per subject [56].

Results are reported as mean ± standard deviation [95% confidence interval (CI)] and as the median and interquartile range (IQR) for the non-normal variables. The significance level was established at *p* ≤ 0.05.

## RESULTS

### External load

The 75%1RM used by the subjects for the squat protocol was 95.33 ± 18.08 kg, 95% CI [87.38–102.97]. As previously mentioned, the number of repetitions for the submaximal sets was established at 9 and 10 with WP and EB, respectively. The number of repetitions for the maximal sets was 10.15 ± 0.81 repetitions, 95% CI [9.85–10.55] for the set with WP and 18.40 ± 4.86 repetitions, 95% CI [16.13–20.67] for the set with EB.

### Ocular variables

The results of the ocular variables are displayed in Table 1. Significant variations were detected in the IOP (F[4,76] = 19.98, *p* < 0.001, ƞp^2^ = 0.513) and MOPP (F[4,76] = 15.13, *p* < 0.001, ƞp^2^ = 0.443) when including the pre-exercise value in the analysis, but not in the CCT (F[4,76] = 0.372, *p* = 0.828, ƞp^2^ = 0.02). Pairwise post-hoc comparisons showed that IOP and MOPP significantly decreased and increased compared to resting levels, respectively. The effect of the material (F[1,19] = 1.78, *p* = 0.19, ƞp^2^ = 0.086), the level of effort (F[1,19] = 1.15, *p* = 0.29, ƞp^2^ = 0.057), and their interaction (F[1,19] = 1.20, *p* = 0.28, ƞp^2^ = 0.060) in the IOP, were non-significant. Similar results were obtained for the MOPP (material: F[1,19] = 0.852, *p* = 0.37, ƞp^2^ = 0.043; level of effort: F[1,19] = 0.039, *p* = 0.84, ƞp^2^ = 0.002; interaction: F[1,19] = 0.069, *p* = 0.79, ƞp^2^ = 0.004).

**TABLE 1:** Ocular outcomes for each of the four squat sets.

Condition	IOP (mmHg)	MOPP (mmHg)	CCT (microns)
Baseline	15.05 ± 3.22*	43.45 ± 5.80*	562.40 ± 29.19
	[13.54–16.56]	[40.74–46.17]	[548.74–576.06]

Set 1 (Max75%1RMWP)	12.85 ± 2.82^(3)^	53.05 ± 8.49	561.15 ± 30.35
	[11.53–14.17]	[49.08–57.03]	[546.95–575.35]
Δ%	14.62	22.09	0.22
Cohen’s *d_unb_*	0.73	1.27	0.04

Set 2 (Submax75%1RMWP)	12.80 ± 2.82	52.59 ± 8.44	562.65 ± 29.50
	[11.48–14.12]	[48.64–56.54]	[548.84–576.46]
Δ%	14.95	21.03	0.04
Cohen’s *d_unb_*	0.74	1.21	< 0.01

Set 3 (Max75%1RMEB)	12.30 ± 2.18^(1)^	51.98 ± 8.34	561.80 ± 28.59
	[11.28–13.32]	[48.07–55.88]	[548.42–575.18]
Δ%	17.95	19.62	0.11
Cohen’s *d_unb_*	1.00	1.14	0.02

Set 4 (Submax75%1RMEB)	12.75 ± 2.55	52.10 ± 6.36	562.00 ± 28.84
	[11.56–13.94]	[49.12–55.07]	[548.50–575.50]
Δ%	15.28	19.89	0.07
Cohen’s *d_unb_*	0.79	1.36	0.01

Note: Values are presented as mean ± standard deviation [95% confidence interval]. Percentage of variation (Δ%) and effect size (Cohen’s d unbiased; *d_unb_* < 0.50 small, 0.50 ≤ *d_unb_* ≤ 0.79 moderate, and *d_unb_* ≤ 0.80 large) compared to baseline values are displayed.

* : Statistically significant difference compared to the rest of the conditions.

1, 2, 3, 4 : Significant difference (*p* < 0.05) or a trend (*p* > 0.05 to *p* < 0.13; if the number is in brackets), with the condition 1, 2, 3, or 4, respectively; Max: maximum number of repetitions; Submax: submaximal repetitions; %1RMWP: percentage of one repetition maximum with weight plates; %1RMEB: percentage of one repetition maximum with elastic bands; IOP: intraocular pressure; MOPP: mean ocular perfusion pressure; CCT: central corneal thickness.

### Cardiovascular parameters and perceived effort

Significant differences were observed in all the cardiovascular variables (PP: F[4,76] = 7.94, *p* < 0.001, ƞp^2^ = 0.295; MBP: F[4,76] = 8.88, *p* < 0.001, ƞp^2^ = 0.318; HR: F[4,76] = 59.44, *p* < 0.001, ƞp^2^ = 0.758). Post-hoc testing revealed that while all the variables significantly increased compared to baseline values, differences among sets only appeared in HR and RPE, as can be seen in Table 2. Regarding the HR, an influence of the level of effort (F[1,19] = 8.12, *p* = 0.01, ƞp^2^ = 0.299) and the interaction material*level of effort (F[1,19] = 5.44, *p* = 0.03, ƞp^2^ = 0.223) was observed, while the influence of the material was non-significant. An influence of the material (F[1,19] = 7.946, *p =* 0.01, ƞp^2^ = 0.295), the level of effort (F[1,19] = 167.83, *p* < 0.001, ƞp^2^ = 0.898), and its interaction (F[1,19] = 15.55, *p* < 0.001, ƞp^2^ = 0.450) was observed on the RPE.

**TABLE 2:** Cardiovascular and perceived effort values for each of the four squat sets.

Condition	PP (mmHg)	MBP (mmHg)	HR (bpm)	RPE
Baseline	58.58 ± 8.04*	87.75 ± 6.97*	63.95 ± 11.27*	-
	[55.16–62.16]	[84.49–91.01]	[59.05–69.21]	

Set 1	73.42 ± 17.40	98.85 ± 11.18	105.26 ± 16.76^(3),4^	8.55 ± 0.88^2,4^
(Max75%1RMWP)	[66.26–81.42]	[93.62–104.08]	[97.95–112.89]	[8.14–8.96]
Δ%	26.37	12.65	65.05	Median 8.5
Cohen’s *d_unb_*	1.05	1.14	2.77	IQR: 1

Set 2	72.25 ± 18.39	98.08 ± 11.66	104.47 ± 17.84^3^	7.55 ± 0.99*
(Submax75%1RMWP)	[63.00–78.47]	[92.63–103.54]	[97.00–112.16]	[7.09–8.01]
Δ%	23.73	11.78	63.09	Median: 7
Cohen’s *d_unb_*	0.80	1.03	2.61	IQR: 1

Set 3	72.20 ± 12.65	96.42 ± 11.97	110.89 ± 23.80^(1),2,4^	8.65 ± 0.93^2,4^
(Max75%1RMEB)	[65.74–76.89]	[90.82–102.02]	[100.53–121.05]	[8.21–9.09]
Δ%	23.64	9.88	74.28	Median: 9
Cohen’s *d_unb_*	1.15	0.85	2.42	IQR: 1

Set 4	73.21 ± 12.68	97.27 ± 8.68	100.47 ± 16.88^1,3^	6.50 ± 1.24*
(Submax75%1RMEB)	[68.21–79.58]	[93.20–101.33]	[93.42–108.26]	[5.92–7.08]
Δ%	25.61	10.85	57.10	Median 6
Cohen’s *d_unb_*	1.32	1.16	2.44	IQR: 1

Note: Values are presented as mean ± standard deviation [95% confidence interval]. Also, median and interquartile range (IQR) are displayed for rate of perceived exertion (RPE) as it is a non-normal variable. Percentage of variation (Δ%) and effect size (Cohen’s d unbiased; *d_unb_* < 0.50 small, 0.50 ≤ *d_unb_* ≤ 0.79 moderate, and *d_unb_* ≤ 0.80 large) compared to baseline values are displayed.

* : Statistically significant difference compared to the rest of the conditions;

1, 2, 3, 4 : Significant difference (*p* < 0.05) or a trend (*p* ≥ 0.05 to *p* < 0.13; if the number is in brackets), with the condition 1, 2, 3, or 4, respectively; Max: maximum number of repetitions; Submax: submaximal repetitions; %1RMWP: percentage of one repetition maximum with weight plates; %1RMEB: percentage of one repetition maximum with elastic bands; PP: pulse pressure; MBP: mean blood pressure; HR: heart rate.

## DISCUSSION

Based on the ocular and systemic responses to a squat exercise protocol, this investigation examined whether elastic bands may modulate these physiological acute adaptations to resistance exercise. Overall, the outcomes of this research were that: 1) IOP significantly decreased and 2) OPP and the cardiovascular values (PP, MBP, HR) significantly increased after all the exercise sets compared to pre-exercise values. Although the study hypothesis could not be confirmed and no effect of the material was observed in the ocular variables, the largest drop in IOP (2.70 mmHg) was observed after a maximal effort with EB at 75%1RM (see Table 1). Empirical evidence was found indicating that EB facilitate a higher number of repetitions (see “Results – External load” section) while maintaining similar pulse pressure and mean blood pressure (see Table 2), which have been related to cardiovascular [33] and ocular health [34]. Also, the rate of perceived effort and HR were not different between performing a mean of 10 repetitions with weight plates and a mean of 18 repetitions with elastic bands both at 75%1RM.

Considering what has been mentioned above, it is worth discussing the outputs of this research under the light of other empirical experiences, which addressed the influence of the external load and other physiological parameters on the IOP. However, this evidence does not contemplate variable resistance as a method of loading the bar.

Squatting with EB reduces the weight at the bottom phases of the squat, during which a mechanical disadvantage occurs [25,26]. Beyond this point, EB add progressively more resistance/weight until the end of the movement. Thus, exercising with EB can increase the load in the region of the range of motion that is more mechanically effective, accompanied by reduced loads in the less efficient range [22,25,26]. This feature of the EB allowed subjects to perform more repetitions, which is useful to promote muscle adaptations [16,27,57]. The combination of all these facts argues in favour of the incorporation of EB in resistance training programmes. Additionally, the use of EB in this study provoked similar values of post-exercise IOP as the use of WP did (maximal effort: mean difference 0.55 mmHg, 95%CI [-0.12-1.22]; *p*=0.10; *d_unb_*=0.210; submaximal effort: mean difference 0.05 mmHg, 95%CI [-0.62-0.72]; *p*=0.87; *d_unb_*=0.018). This combination of findings suggests that EB is an appropriate device to load the bar for squat exercises when looking for high volumes and conservative ocular responses. To support these findings, independent variables included in our study and their influence on IOP are discussed below.

The independent variable of the external load addressed by the expert literature as having major relevance for the changes in ocular physiology is the intensity (weight). Most of the current literature reports higher IOP values with higher intensities [6,7,9,14,16,17,58]. Oppositely to the intensity, a higher volume of repetitions has been related to lower IOP values [6,8,14,16]. In our study, the set with a larger number of repetitions (maximum effort at 75%1RM with EB) provoked the lowest IOP (12.30 mmHg), although the differences with the rest of the sets were not significant. Supporting the influence of the volume on IOP, differences were not found between performing 9 and 10.15 repetitions with WP or between 9 and 10 repetitions with WP and EB, respectively. Lower IOP values in response to a larger number of repetitions may be due to physiological acute adaptations to higher volumes of exercise, such as modifications in plasma pressures, biochemical responses [2–4,27], and higher levels of blood lactate [16,59]. Understanding that lower intensities allow for higher volumes (and higher intensities for lower volumes) [60], it is important to address the possible influence of the level of effort within the ocular acute adaptations to resistance exercise.

The level of effort (i.e. number of repetitions performed out of the maximum possible) has been shown to influence IOP and OPP behaviour, with certain controversy regarding the safety of performing maximal efforts [8,12,16]. While one study reported reductions in IOP after maximum number of repetitions at 60%1RM [16], other authors recommended not including maximal efforts when looking forward to avoiding IOP increases and OPP decreases [8]. Our results indicate that no difference existed between performing a maximal or submaximal effort at 75%1RM with WP (m.d. 0.05 mmHg, 95%CI [-0.59-0.69], *p*=0.87, *d_unb_*=0.017) or EB (m.d. 0.45 mmHg, 95%CI [-0.15-1.05], *p*=0.13, *d_unb_*=0.18).

All cardiovascular values were significantly modified from pre-exercise values. However, differences between sets only emerged in RPE and HR; no influence of the EB compared to WP was observed for the BP values (MBP and PP), as happened with the MOPP. Bearing in mind these results and while caution should be applied, resistance training with elastic bands could be performed by people with hypertension risk just as it can be performed with WP [61,62]. It must be noted that the set which caused the highest HR (maximal effort at 75%1RM with EB), coinciding with the largest volume of repetitions, was also the set with the lowest IOP values, although, and as previously mentioned, the differences were not significant. This fact is probably related to the total volume of work, which increases HR [63] and modulates IOP responses [2–4]. As could be expected, the maximal sets were perceived as harder than the sub-maximal sets. It is also worth highlighting that a maximal effort at 75%1RM with EB was not perceived as harder than with WP, while the set with EB presented a greater number of repetitions.

All the aforementioned variations may be due to different physiological, homeostatic mechanisms. First, we can state that IOP variations were not mediated by CCT changes, as this variable remained stable within all the conditions. This confirms the findings of Read & Collins [64] on stable CCT values after moderate-intensity bicycle ergometer exercise and supports the hypothesis of Wylęgała et al. [3] that the increases reported in CCT after high-altitude climbing might be attributed to low oxygen concentration at higher altitudes rather than to the exercise effect. As for the OPP, exercise-induced changes seem to be mediated by the BP rather than by the IOP, which confirms previous research on the relationship between OPP and BP [2–4,34,65].

### Limitations and future directions

Although all the procedures and analyses were carefully designed and carried out, some limitations should be listed. Firstly, validated air-puff tonometry was chosen as it is easy to use, and does not require the use of anaesthesia [46,47]. However, future studies should standardize a method of continuous IOP measurement [66] and thus, this study should be replicated continuously monitoring the IOP during the squats and for a prolonged period after the exercise to perform a daily curve; diagnosed or suspected glaucoma patients and elderly subjects should be included with a greater sample size. Also, and even though the order of the sets was randomized (across subject counterbalance), future studies should test all the sets in a complete counterbalanced order to be able to determine the effects of the order of exercises on the ocular responses. BP and OPP were estimated with formulas as direct measurements have been proven difficult to carry out in laboratory practices [65]. However, HR significantly changed between sets and this may modulate the fraction of systole (which is used in the MBP formula as the constant) [67]. Also, a method to equalise the weight between EB and WP, as proposed in recent expert literature [22], would help to better understand ocular responses to both materials. It would be interesting to evaluate the kilograms used throughout the range of motion with EB to compare the effects of using the same mean weight with both materials. In this regard, in different pilot studies we performed, we found a descent in the load of about 40% from the standing position to the lowest point of the execution (in our pilot studies located at 81.12±3.74 knee joint angle degrees). Finally, future studies should apply different intensities and account for differences in muscle activation between WP and EB.

### Practical implications

The evidence presented highlights the potential practical applications of using elastic bands to achieve a higher number of repetitions while maintaining safe levels in the analysed ocular health-related and cardiac parameters. We could encourage the professionals interacting with people with glaucoma risk factors to instruct their clients to control the technique, movement tempo, and avoid the Valsalva manoeuvre. Bearing this in mind, both materials studied (elastic bands and weight plates) could be indistinctly used depending on the aims of the training programme without obtaining significant differences in the ocular variables analysed.

## CONCLUSIONS

The most notable finding was that, although the elastic bands allow for more repetitions and add less resistance in the lowest phases of the range of motion than the weight plates, the intraocular pressure, mean ocular perfusion pressure, pulse pressure, and mean blood pressure did not significantly differ. Data from this study indicate that post-exercise IOP is lower, and MOPP higher compared with resting values, after maximal or submaximal efforts, and both with EB and WP. This combination of findings provides some support for the usefulness of EB to perform resistance exercises and, while further research is needed in this regard, suggests that ocular health can be preserved with their use. This research contributes to the multidisciplinary collaboration between optometrists, ophthalmologists, and strength and conditioning professionals for the management and prevention of glaucoma.
